# Prediction of osteoporosis using radiomics analysis derived from single source dual energy CT

**DOI:** 10.1186/s12891-022-06096-w

**Published:** 2023-02-07

**Authors:** Jinling Wang, Shuwei Zhou, Suping Chen, Yewen He, Hui Gao, Luyou Yan, Xiaoli Hu, Ping Li, Hongrong Shen, Muqing Luo, Tian You, Jianyu Li, Zeya Zhong, Kun Zhang

**Affiliations:** 1 Department of Radiology, The First Hospital of Hunan University of Chinese Medicine, 95 Shaoshan Middle Road, Yuhua District, Changsha, 410007 People’s Republic of China; 2 College of Integrated Traditional Chinese and Western Medicine, Hunan University of Chinese Medicine, 300 Xueshi Road, Yuelu District, Changsha, 410208 People’s Republic of China; 3 GE Healthcare (Shanghai) Co., Ltd., Shanghai, 201203 People’s Republic of China

**Keywords:** Osteoporosis, Radiomics, Lumbar vertebrae, Single source dual-energy CT

## Abstract

**Background:**

With the aging population of society, the incidence rate of osteoporosis is increasing year by year. Early diagnosis of osteoporosis plays a significant role in the progress of disease prevention. As newly developed technology, computed tomography (CT) radiomics could discover radiomic features difficult to recognize visually, providing convenient, comprehensive and accurate osteoporosis diagnosis. This study aimed to develop and validate a clinical-radiomics model based on the monochromatic imaging of single source dual-energy CT for osteoporosis prediction.

**Methods:**

One hundred sixty-four participants who underwent both single source dual-energy CT and quantitative computed tomography (QCT) lumbar-spine examination were enrolled in a study cohort including training datasets (*n* = 114 [30 osteoporosis and 84 non-osteoporosis]) and validation datasets (*n* = 50 [12 osteoporosis and 38 non-osteoporosis]). One hundred seven radiomics features were extracted from 70-keV monochromatic CT images. With QCT as the reference standard, a radiomics signature was built by using least absolute shrinkage and selection operator (LASSO) regression on the basis of reproducible features. A clinical-radiomics model was constructed by incorporating the radiomics signature and a significant clinical predictor (age) using multivariate logistic regression analysis. Model performance was assessed by its calibration, discrimination and clinical usefulness.

**Results:**

The radiomics signature comprised 14 selected features and showed good calibration and discrimination in both training and validation cohorts. The clinical-radiomics model, which incorporated the radiomics signature and a significant clinical predictor (age), also showed good discrimination, with an area under the receiver operating characteristic curve (AUC) of 0.938 (95% confidence interval, 0.903–0.952) in the training cohort and an AUC of 0.988 (95% confidence interval, 0.967–0.998) in the validation cohort, and good calibration. The clinical-radiomics model stratified participants into groups with osteoporosis and non-osteoporosis with an accuracy of 94.0% in the validation cohort. Decision curve analysis (DCA) demonstrated that the radiomics signature and the clinical-radiomics model were clinically useful.

**Conclusions:**

The clinical-radiomics model incorporating the radiomics signature and a clinical parameter had a good ability to predict osteoporosis based on dual-energy CT monoenergetic imaging.

**Supplementary Information:**

The online version contains supplementary material available at 10.1186/s12891-022-06096-w.

## Introduction

Osteoporosis is a commonly encountered disease, and is characterized by decreased bone mineral density (BMD) and a damaged bone microstructure, resulting in an increased bone fragility and fracture susceptibility [1]. With the aging population of society, osteoporosis and osteoporosis-related fractures are leading causes of morbidity and mortality in the elderly [2, 3]. Hence, the early diagnosis of osteoporosis plays a significant role in the progress of disease prevention.

Currently, BMD measurement is considered clinically as an important means in the early diagnosis, prevention, and treatment of osteoporosis [4]. The reference standards for the BMD measurement include double-energy X-ray absorptiometry (DXA) and quantitative computed tomography (QCT) [5, 6]. DXA is used to measure the mean values of areal BMD (in g/cm^2^) for cortical bone and trabecular bone [7], whereas the accuracy of estimated bone mass value is limited by the inherent interference derived from hyperosteogeny, sclerosis, et al. [8, 9]. As a recognized approach for volumetric BMD (in mg/cm^3^) assessment of specifically trabecular bone [10], QCT holds a higher sensitivity on osteoporosis prediction than DXA [11–14], however, the application of QCT remains limited by the complex post-processing (extra calibration and standardized software) as well as higher radiation dose. In recent years, radiomics introduces a new quantitative approach for disease assessment. Based on the acquired radiography, computed tomography (CT) or magnetic resonance (MR) images, it applies advanced data model algorithms to extract high-throughput imaging features, and find radiomics features that are difficult to identify visually, so as to provide accurate disease diagnosis, malignant tumor classification, prognosis prediction and efficacy evaluation. Moreover, radiomics is more comprehensive and convenient, without adding additional radiation dose [15–18].

However, for conventional CT radiomics-based osteoporosis prediction, there are challenges in providing sufficient accuracy owing to the polychromatic images and beam-hardening artifacts. With the advances in technologies, dual-energy CT might be the solution. Derived from single source dual-energy CT, virtual monochromatic spectral (VMS) images are reconstructed from a pair of accurate material density images and mass attenuation coefficients. VMS imaging can yield improved image quality by reducing beam-hardening artifacts [19]. Single source dual-energy CT provides 101 sets of 40–140 keV VMS images for different clinical needs, resulting in more precise measurement of CT value which constitute radiomic features. Previous studies have shown that VMS images at approximately 70 keV (range, 67–72 keV) had lower image noise and a higher contrast-to-noise ratio (CNR) than 120-kVp CT images [20, 21]. Moreover, the radiation exposure dose of single source dual-energy CT with the volume-based adaptive statistical iterative reconstruction (ASiR-V) technique is equal to or lower than that of conventional CT [22–24]. But, reports on the use of radiomics analysis derived from single source dual-energy CT for osteoporosis prediction is still lacking.

Therefore, the purpose of our study was to explore the value of radiomics analysis based on the monochromatic imaging of single source dual-energy CT for osteoporosis prediction, with QCT referenced.

## Methods

### Study participants

**Fig. 1:**
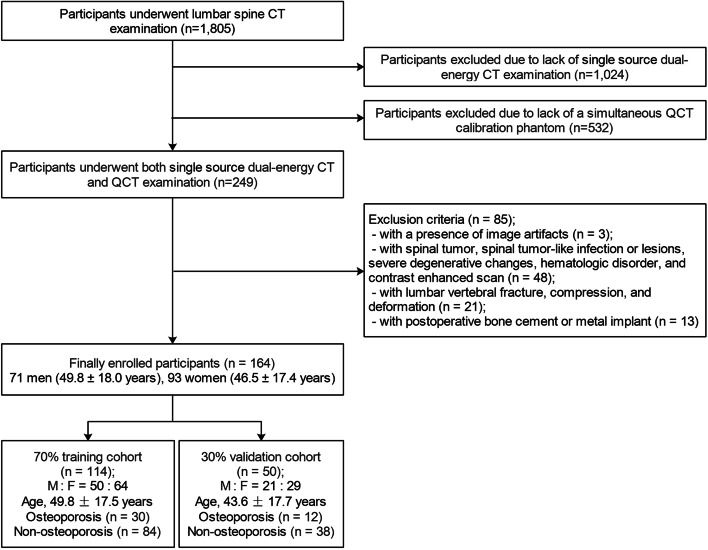
Flowchart of participants enrollment and random selection for radiomics analysis

The institutional ethics committee of The First Affiliated Hospital of Hunan University of Chinese Medicine approved this retrospective study and the requirement to obtain informed consent from all participants was waived (no. HN-LL-KY-2021–019-01), the data were desensitized before using in order to protect the participant’s privacy. Data from participants who underwent both single source dual-energy CT and QCT lumbar-spine examination between September 2019 and August 2020 were collected. Clinical data including age, sex, and body mass index (BMI) were recorded before scanning. Participants were excluded if they were (a) with a presence of image artifacts; (b) with spinal tumor, spinal tumor-like infection or lesions, severe degenerative changes, hematologic disorder, and contrast enhanced scan; (c) with lumbar vertebral fracture, compression, and deformation; (d) with postoperative bone cement or metal implant [25]. Finally, a total of 164 participants [71 men (mean age: 49.8 ± 18.0 years, range: 22—87 years) and 93 women (mean age: 46.5 ± 17.4 years, range: 23—90 years)] were enrolled in the study. Participants were randomly allocated to training and validation cohorts in a 7: 3 ratio. The flowchart of participants selection is showed in Fig. 1. With QCT as the reference standard, diagnosis was performed according to the guidelines introduced by the International Society for Clinical Densitometry (ISCD) and American College of Radiology (ACR), osteoporosis of the spine was defined as a BMD value < 80 mg/cm^3^, and non-osteoporosis (osteopenia and normal) was defined as a BMD value ≥ 80 mg/cm^3^ [26].

### Single source dual-energy CT and phantom-calibrated QCT imaging acquisition

In order to avoid additional radiation exposure and substantial costs in our study, all lumbar-spine examinations of the enrolled participants were simultaneously performed with a bone density calibration (BDC) phantom placed beneath the spine. CT examinations were performed on a single source dual-energy CT scanner (Revolution, GE Medical Systems, Milwaukee, WI, USA). The scan parameters were as follows: GSI helical mode with fast kilovoltage (KV)-switching between 80 and 140 kVp; tube current, 230 mA; beam collimation, 128 × 0.625 mm; rotation speed, 0.8 s/rot; helical pitch, 0.984; bone kernel; and 50% ASiR-V. Then, a series of contiguous 1.25-mm-thick VMS images at 70 keV were generated for subsequent BMD measurement and radiomics analysis [21]. The details of BMD measurement by phantom-calibrated QCT refer to our previous article [25].

### Image segmentation

**Fig. 2:**
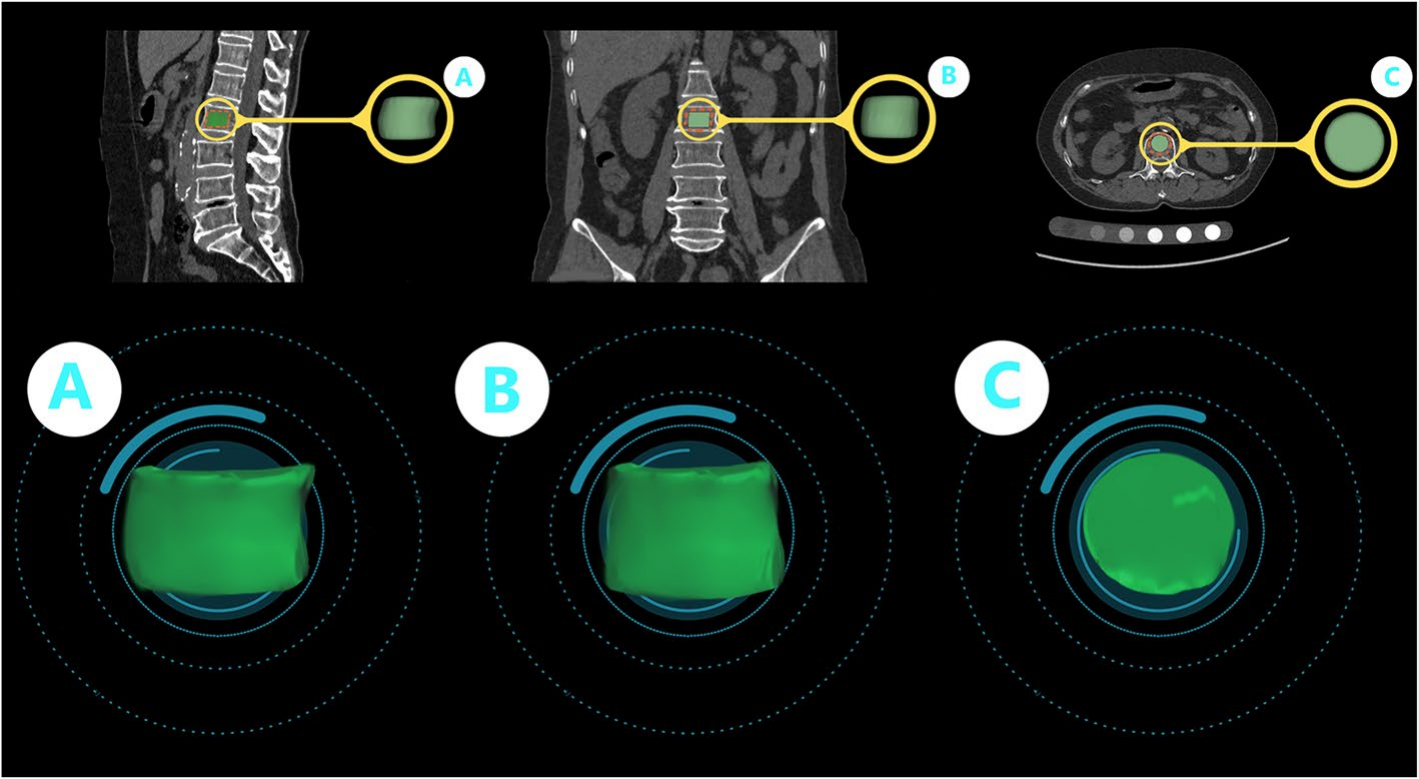
Representative volume-of-interest (VOI) in the 70-kev monochromatic image of the lumbar spine CT of an osteoporotic participant. **A**, **B**, **C**, VOIs on sagittal, coronal and axial CT images, respectively

The volume-of-interest (VOI) was manually drawn on the axial 70-keV monochromatic images from single source dual-energy CT by reader 1 (L Yan, with 4 years of experience in medial image segmentation) using the 3D-Slicer software (version 4.11.20200930; http://www.slicer.org). The VOI with a diameter of 20 mm and a height of 12.5 mm was acquired in the middle of the L1-L5 vertebral bodies on eleven consecutive images. Typically, the boundary was set along the inner edge of vertebral cortex, and the areas of the cortical bone and the vertebral venous plexuses posteriorly were carefully avoided (Fig. 2). To assess interobserver variability in the segmentation, 33 participants were randomly selected and were manually segmented by reader 2 (Y He, with 2 years of experience in medial image segmentation) in the same way. The image segmentation was supervised by a radiologist (K Zhang, with 13 years of experience in musculoskeletal radiology).

### Radiomics feature extraction

PyRadiomics [27], an open-source python package recommended for standardized radiomics analysis workflow [28], was used to extract 107 radiomics features from each VOI. These 107 extracted features were designed to characterize the VOIs in seven parts: 18 first-order statistics features, 14 shape features, 24 Gy level co-occurrence matrix (GLCM) features, 16 Gy level size zone matrix (GLSZM) features, 16 Gy level run length matrix (GLRLM) features, 5 neighboring gray tone difference matrix (NGTDM) features, and 14 Gy level dependence matrix (GLDM) features. All images were resampled to a voxel size of 1 × 1 × 1 mm to standardize the voxel spacing, and were quantized with a quantization range of mean ± 3 × SD and 25 bins without filtering.

Details of the radiomics features are described (Supplementary Data S1).

### Selection of radiomics features

The following three steps were undertaken to select robust radiomics features.

First, the intraclass correlation coefficients between the VOI-based radiomics features extracted from multiple cases (the cases of 33 randomly selected and segmented by reader 1 and reader 2) were obtained to determine reproducibility. Stable features with an intraclass correlation coefficient (ICC) > 0.90 were accepted and were included for subsequent analysis.

Second, to further reduce dimensionality, the univariate statistical tests (two-sample t test) between osteoporosis and non-osteoporosis groups were examined, and features were retained with *p* < 0.05.

Finally, the least absolute shrinkage and selection operator (LASSO) regression [29] was performed with penalty parameter tuning using five fold cross-validation, and features with non-zero coefficients were selected.

### Models construction and evaluation

#### Radiomics model

A radiomics signature was constructed from linear combinations of the selected features weighted by their respective LASSO coefficients.

#### Clinical-radiomics model

For each of the four variables (radiomics signature and three clinical parameters [age, sex, BMI]), the univariate logistic regression analysis was performed. Significant parameters from the univariate analysis were selected and were included in the multivariate logistic regression analysis to construct a clinical-radiomics model.

The performance of the two models was assessed in terms of calibration and discrimination. Calibration was quantified with the Hosmer Lemeshow goodness-of-fit test. Discrimination was assessed with the area under the receiver operating characteristics (ROC) curve. The optimal cutoff values for classifying participants according to osteoporosis or non-osteoporosis were determined on the highest Youden index (sensitivity + specificity—1) value in the ROC curves of the training cohort. Accuracy, specificity, sensitivity, negative predictive value (NPV), and positive predictive value (PPV) were obtained.

The performance of the two models was then tested in the validation cohort.

### Clinical use

Decision curve analysis (DCA) was conducted to estimate the clinical usefulness of the radiomics signature and the clinical-radiomics model by calculating the net benefits for a range of threshold probabilities in the validation cohort.

### Statistical analysis

All statistical analyses were performed with IBM SPSS Statistics 25 and R software (version 3.6.1, https://www.r-project.org). Student or the Mann–Whitney test was utilized to analyze continuous variables, and the chi-square or Fisher’s exact test was used to analyze the differences between the groups in categorical variables. *P* values < 0.05 were considered statistically significant.

## Results

### Demographic and clinical characteristics

The demographic and clinical characteristics of osteoporosis and non-osteoporosis participants are summarized in Table 1. There were 114 participants in the training cohort (30 osteoporosis and 84 non-osteoporosis) and 50 participants in the validation cohort (12 osteoporosis and 38 non-osteoporosis). There was no significant difference in participants’ demographic and clinical characteristics between the training and validation datasets (Table 2).

**Table 1:** Demographic and clinical characteristics of osteoporosis and non-osteoporosis participants

Variables	Osteoporosis (*n* = 42)	Non-osteoporosis (*n* = 122)	*P* value
Age (years)^a^	67.12 ± 10.65	41.28 ± 14.53	< 0.000
sex (M:F)	14:28	57:65	0.131
BMI (kg/m^2^)^a^	22.47 ± 3.78	22.51 ± 3.21	0.951

*BMI* Body mass index

^a^Mean ± standard deviation

**Table 2:** Participants’ demographic and clinical characteristics in the training and validation cohorts

	Training (*n* = 114)			Validation (*n* = 50)			
	Osteoporosis (*n* = 30)	Non-osteoporosis (*n* = 84)	*P* value	Osteoporosis (*n* = 12)	Non-osteoporosis (*n* = 38)	*P* value	*P* value^*^
Age (years)^a^	67.50 ± 11.44	43.45 ± 14.66	< 0.000	66.17 ± 8.72	36.47 ± 13.18	< 0.000	0.061
sex (M:F)	11:19	39:45	0.355	3:9	18:20	0.302	0.825
BMI (kg/m^2^)^a^	23.22 ± 3.81	22.23 ± 3.19	0.169	20.61 ± 3.11	23.13 ± 3.30	0.021	0.951
Osteoporosis (O:N)	30:84			12:38			0.754

*BMI* Body mass index, *O* Osteoporosis, *N* Non-osteoporosis

^a^Mean ± standard deviation

^*^Difference between training and validation cohorts

### Radiomics feature selection

**Fig. 3:**
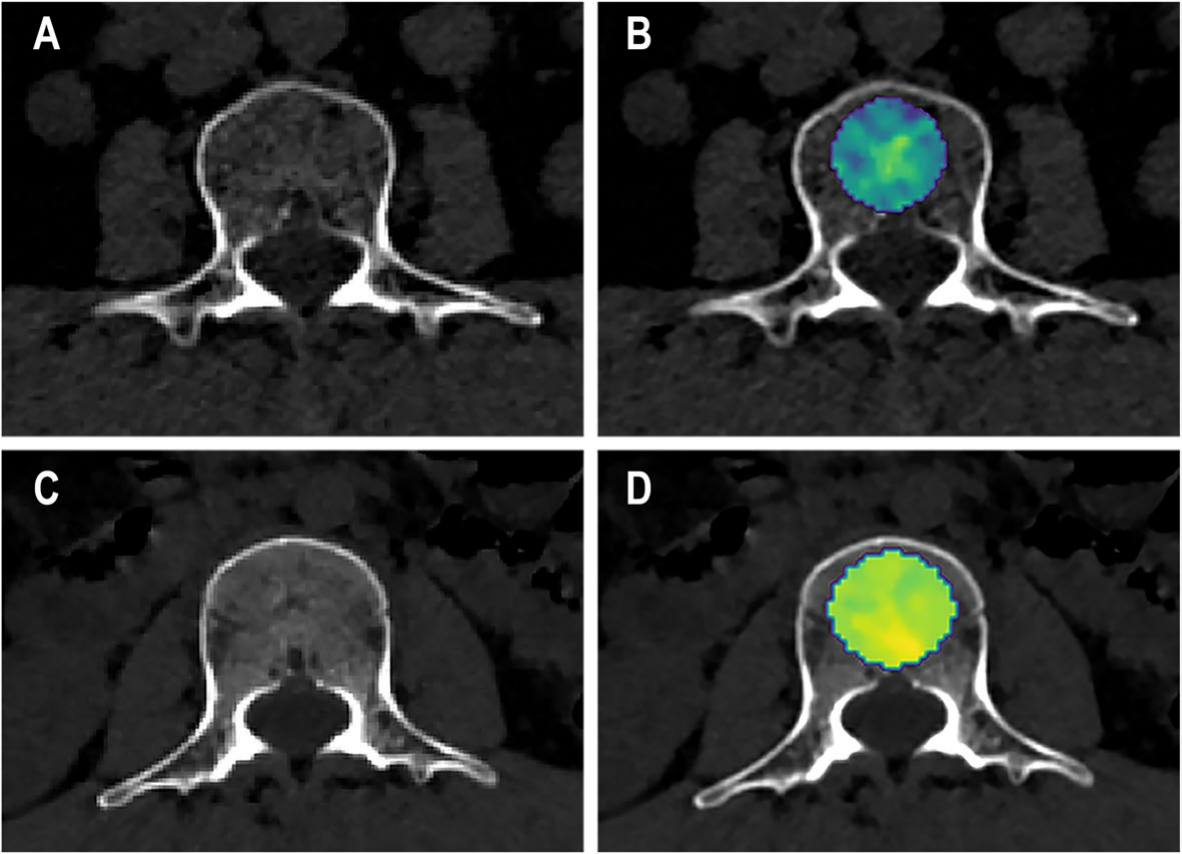
Radiomics analysis in an osteoporotic participant at L3 body (**A**-**B**) and a non-osteoporotic participant at L3 body (**C**-**D**). Illustrated is the original_firstorder_Mean feature overlaid on the axial CT images (**B**, **D**)

Among the 107 radiomics features, 71 stable features were identified with an ICC > 0.90 and were included for subsequent analysis (Supplementary Data S2). Then, these 71 features were further reduced to 23 features by applying the two-sample t test. Finally, the LASSO regression model led to the selection of 14 features with non-zero coefficients, which consisted of seven first-order statistics features and seven texture features (Table 3; Fig. 3).

**Table 3:** The eleven radiomics features used to develop the radiomics signature and their respective coefficients

Feature type	Feature name	LASSO coefficient (β)
First-order	original_firstorder_90Percentile	0.090
First-order	original_firstorder_Maximum	0.121
First-order	original_firstorder_Mean	0.523
First-order	original_firstorder_Median	0.147
First-order	original_firstorder_Minimum	0.081
First-order	original_firstorder_Skewness	-0.044
First-order	original_firstorder_TotalEnergy	-0.493
GLCM	original_glcm_Idmn	0.023
GLDM	original_gldm_LargeDependenceHighGrayLevelEmphasis	-0.026
GLDM	original_gldm_SmallDependenceLowGrayLevelEmphasis	-0.047
GLSZM	original_glszm_LargeAreaLowGrayLevelEmphasis	-0.021
GLSZM	original_glszm_LowGrayLevelZoneEmphasis	0.018
GLSZM	original_glszm_SmallAreaLowGrayLevelEmphasis	0.026
NGTDM	original_ngtdm_Busyness	0.005

*GLCM* Gray level co-occurrence matrix, *GLDM* Gray level dependence matrix, *GLSZM* Gray level size zone matrix, *NGTDM* Neighboring gray tone difference matrix

### Construction, performance, and validation of the models

For radiomics model, the radiomics signature was constructed from the 14 robust features as follows:

Radiomics signature = 1.453 + (0.090 ∙ 90th percentile) + (0.121 ∙ maximum) + (0.523 ∙ mean) + (0.147 ∙ median) + (0.081 ∙ minimum) + (-0.044 ∙ skewness) + (-0.493 ∙ total energy) + (0.023 ∙ idmn) + (-0.026 ∙ large dependence high gray level emphasis) + (-0.047 ∙ small dependence low gray level emphasis) + (-0.021 ∙ large area low gray level emphasis) + (0.018 ∙ low gray level zone emphasis) + (0.026 ∙ small area low gray level emphasis) + (0.005 ∙ busyness).

For clinical-radiomics model, the results of the univariate and multivariate analyses are summarized in Table 4. Among the clinical parameters, age was found to be a significant predictor. A clinical-radiomics model was constructed based on the radiomics signature and a significant clinical predictor as follows:

**Table 4:** Results of univariate and multivariate logistic regression analysis of potential predictors of osteoporosis

	Univariate		Multivariate		
			Clinical-radiomics model		
	OR (95% CI)	*p* value	Beta	OR (95% CI)	*p* value
Radiomics signatures	3.749(2.453,5.728)	< 0.000^*^	1.143	3.137(1.892,5.202)	< 0.000^*^
Age^a^	0.875(0.838,0.914)	< 0.000^*^	-0.094	0.911(0.870,0.953)	< 0.000^*^
sex	1.334(0.651,2.734)	0.431			
BMI	1.003(0.903,1.114)	0.951			

*OR* Odds ratio

^a^Per 1-year increment

^*^Statistically significant (*P* < 0.05)

$\mathrm{ln}\left[\mathrm{P}/\left(1-\mathrm{P}\right)\right]$ = 4.989 + (1.143 ∙ radiomics signature) + (-0.094 ∙ age), where $\mathrm{P}$ is the predicted probability of osteoporosis.

**Fig. 4:**
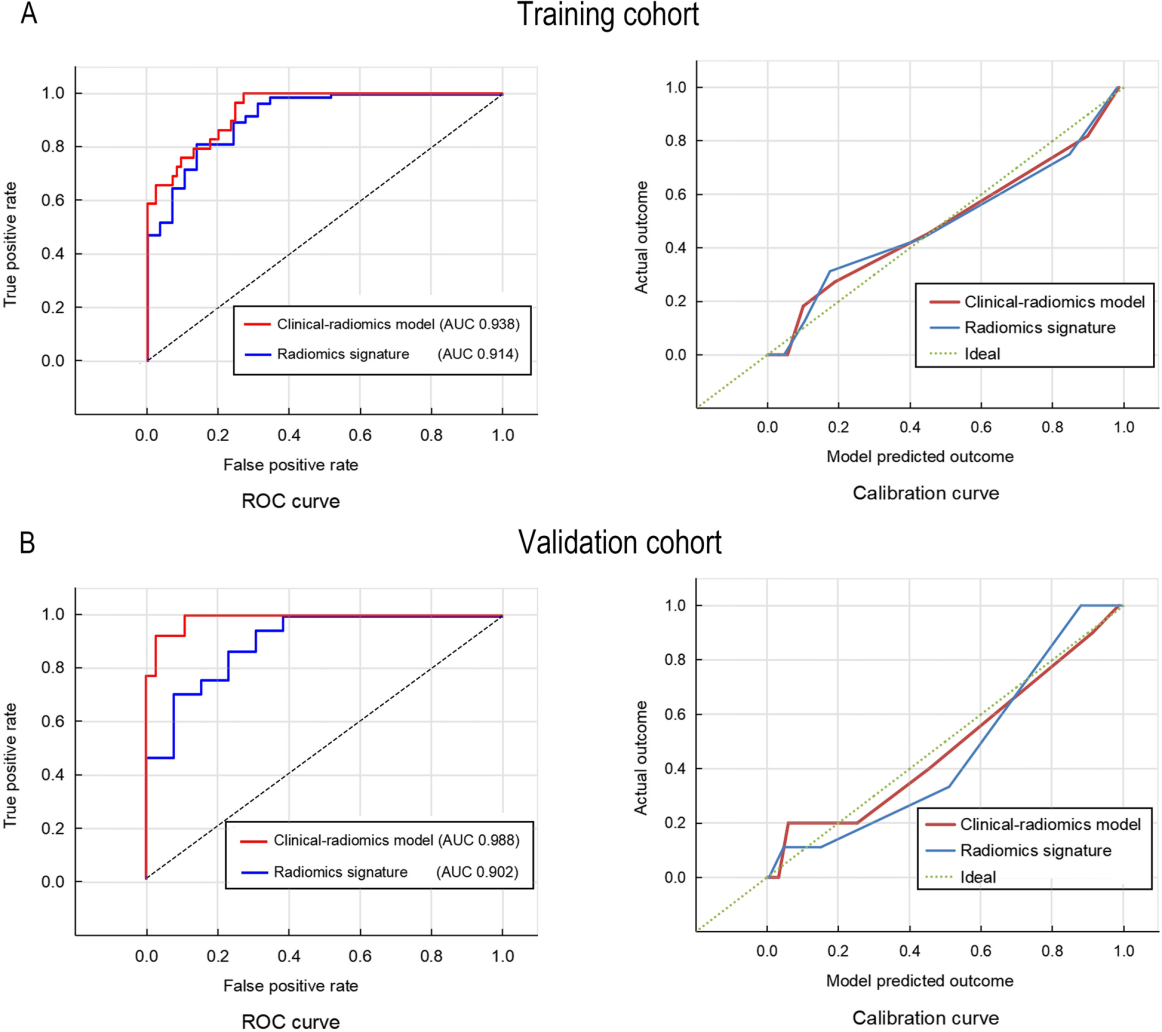
Receiver operating characteristic curves and calibration curves of the clinical-radiomics model and the radiomics signature for the prediction of osteoporosis in the (**A**) training and (**B**) validation cohorts

The diagnostic performance of the above two models is summarized in Table 5, and the receiver operating characteristic curves and calibration curves were plotted (Fig. 4). The clinical-radiomics model showed good discrimination (AUC, 0.938 in training cohort and 0.988 in validation cohort), and calibration according to the Hosmer–Lemeshow test in both training (*p* = 0.880) and validation (*p* = 0.905) cohorts. The radiomics signature also showed good discrimination and calibration performance in both training (AUC, 0.914; Hosmer–Lemeshow test, *p* = 0.810) and validation (AUC, 0.902; Hosmer–Lemeshow test, *p* = 0.758) cohorts.

**Table 5:** Diagnostic performance and validation of prediction models

	Radiomics signature		Clinical-radiomics model		
	Training	Validation	Training	Validation	*P* value^*^
AUC^a^	0.914(0.892,0.931)	0.902(0.880,0.928)	0.938(0.903,0.952)	0.988(0.967,0.998)	0.643
Cutoff	1.05	0.09	-1.46	0.15	
Accuracy (%)	86.8	86.0	88.6	94.0	
Specificity (%)	75.0	80.0	89.1	97.2	
Sensitivity (%)	90.7	87.5	86.4	85.7	
NPV (%)	72.4	61.5	96.5	94.6	
PPV (%)	91.8	94.6	65.5	92.3	

*AUC* Area under the receiver operating characteristics curve, *NPV* Negative predictive value, *PPV* Positive predictive value

^a^Data in parentheses are 95% confidence interval

^*^Difference between training and validation cohorts

### Clinical use

**Fig. 5:**
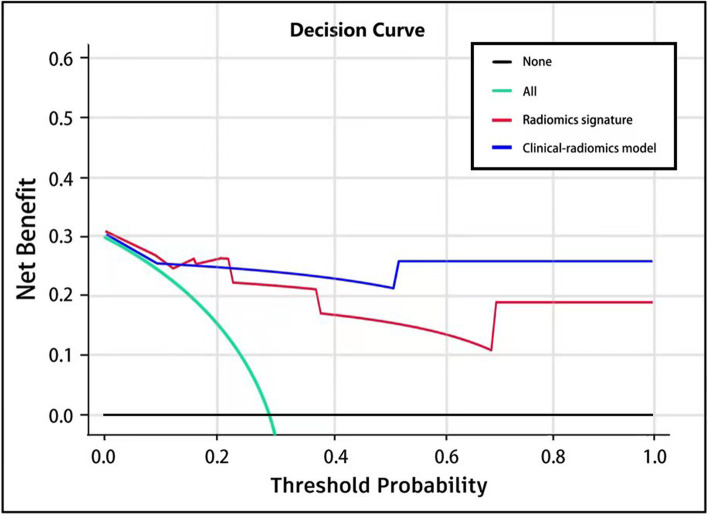
Decision curve for the radiomics signature and the clinical-radiomics model

The DCA result for the two models is shown in Fig. 5. The decision curve showed that if the threshold probability of a doctor or patient is > 10%, using the radiomics signature and the clinical-radiomics model to predict osteoporosis leads to a higher overall net benefit than the treat-none and treat-all-patients schemes.

## Discussion

In our study, we developed and validated two models (radiomics signature and clinical-radiomics model) based on the monochromatic imaging of single source dual-energy CT for osteoporosis prediction. The proposed clinical-radiomics model, incorporating the radiomics signature and a significant clinical predictor (age), stratified participants into osteoporosis and non-osteoporosis groups with higher accuracies (88.6% in training and 94.0% in validation cohorts) than the radiomics signature alone.

A recent study has demonstrated that a deep learning-based method could achieve fully automatic identification of vertebral osteoporosis, osteopenia, and normal bone mineral density in 120 kVp polychromatic CT images [30]. In fact, the true single-energy CT value is defined as the attenuation value after the monochromatic X-rays passing through the detected tissue. But, in conventional single-source CT, the polychromaticity of the X-rays commonly causes averaging attenuation effect and beam-hardening artifacts in the image, and leads to inaccurate measurement of CT values [31], which may have a potential effect on the accuracy of the deep learning-based study. The recently developed dual-energy CT imaging uses single source and fast kVp-switching between 80 and 140 kVp to simulate the imaging of materials under monochromatic X-ray source by analyzing the information during dual-energy data acquisition to produce VMS images. By doing so, single source dual-energy CT could greatly reduce beam-hardening artifacts and generate images with more accurate CT attenuation values at every energy level. Moreover, in previous studies [20], the approximately 70 keV monochromatic image has characterized by lower image noise and a higher CNR, and has potentially replaced the 120 kVp polychromatic image used in conventional image processing. It also reported that the 70 keV monochromatic image had the lowest artifact index among all energy levels [31]. So, in our study, the 70 keV monochrome image with improved image quality was selected thus looked forward to better diagnostic efficiency.

Several studies have applied radiomics to predict osteoporosis. He et al. proposed the classification method of normal vs. osteopenia, normal vs. osteoporosis, and osteopenia vs. osteoporosis using radiomics based on T1-wighted and T2-wighted sagittal lumbar spine magnetic resonance (MR) images, and the AUC was 0.810, 0.797, and 0.769, respectively [32]. Compared with the results of their study, our models applied to CT images showed superior discrimination performance. In one recent study by Lim et al., abdomen-pelvic CT (APCT)-based radiomics analysis with a machine learning algorithm was used to predict femoral osteoporosis [33]. In their study, a machine learning classifier could classify participants into osteoporosis and non-osteoporosis with accuracy of 92.9% and 92.7% in the training and validation cohorts, respectively, indicating radiomics approach based on CT images has good potential for osteoporosis prediction. In our study, to enhance robustness, we employed similar method to select reproducible features from multiple cases (ICC > 0.90), and our models achieved a comparable accuracy for vertebral osteoporosis prediction. Further, in our study, to develop a more holistic model, we synchronously incorporated the radiomics signature and a significant clinical predictor (age). In line with previous studies, the ages of the participants with osteoporosis were significantly older than those with non-osteoporosis [34, 35].

Most previous studies [32, 33, 36] had a common problem that the areal BMD (in g/cm^2^) measured by DXA was considered as the reference standard, which may have a potential effect on the accuracy of these studies. It is well known that QCT can measure true volumetric BMD (in mg/cm^3^) and provide more accurate BMD values than DXA. Moreover, there may have better agreement between volumetric BMD and the radiomic features of VOIs. To date, few studies on radiomics of osteoporosis have used QCT as the reference standard. In our study, with QCT as reference, the proposed models had a good ability to predict osteoporosis, showing that QCT and VOIs are more corresponding. Radiomics analysis may be a new approach for the diagnosis of osteoporosis.

Radiomics features are quantitatively extracted agnostic features that are considered to reflect intra-region heterogeneity [37]. Our radiomics signature included seven first order statistics features and seven texture features, respectively describing the distribution and spatial arrangement of voxel intensities within the VOI. Among the fourteen features, original_firstorder_Mean was the most predictive feature with the largest LASSO coefficient, representing the average gray level intensity within the VOI. Several studies [38] have shown a significant association between BMD-decrease and the first order statistics change, including mean CT Hounsfield unit (HU). Moreover, some complex texture features have been reported to be linked to known pathological changes in the osteoporotic bone, such as GLCM features [39–42]. In our study, we found that osteoporosis had a lower mean gray level intensity than non-osteoporosis, which should be highly related to the processes that occur in osteoporosis. Osteoporosis causes bone mineral loss and destruction of the delicate bony microstructure especially trabecular bone which is more active metabolically [43]. Therefore, we speculated that osteoporosis would show lower mean intensity of gray levels within a VOI due to decreased bone calcium and increased fatty marrow than non-osteoporosis. These subtle differences in statistics and pathology reflect the difference in heterogeneity of these two groups, which are difficult to identify by visual assessment of CT images.

This study has several limitations that should be noted. First, as a retrospective study with a relatively small sample size, there is a possibility of participant selection bias in our cohorts. Second, the proposed models were established on the basis of data obtained from a single center and single race. Therefore, it is our future work to prove the validity through prospective multi-center and multi-ethnic studies. Third, our study did not directly show the advantage of using dual-energy CT monochromatic images in constructing radiomics signature over the conventional kVp images. Finally, our study cohort was divided into two groups: osteoporosis and non-osteoporosis groups, which consist of normal and osteopenia participants. And this was due to the purpose of our study was to develop and validate models based on the monochromatic imaging of single source dual-energy CT for osteoporosis prediction. Thus, this study was focused on osteoporosis prediction. In the future, we will aim to evaluate whether radiomics analysis is possible to predict osteoporosis, osteopenia, and normal status accurately.

In conclusion, our proposed models, with QCT regarded as the reference standard and the VOI-based radiomics features extracted from the 70 keV monochromatic images, had a good ability to predict osteoporosis. The predictive models could serve as potential decision support tools for both radiologists and clinicians.

## Supplementary Information

**Additional file 1.**

## Data Availability

The datasets in this study are available on request from the corresponding author. The datasets are not publicly available due to privacy or ethical restrictions.
