# Supplementary material for: Prediction of osteoporosis using radiomics analysis derived from single source dual energy CT
Source: BMC Musculoskelet Disord. 2023 Feb 7;24:100. doi: 10.1186/s12891-022-06096-w (PMC9903590; doi:10.1186/s12891-022-06096-w)
Supplement: Supplementary file 1 — Additional file 1. [file 12891_2022_6096_MOESM1_ESM.docx]

**Supplementary Data S1.**

List of the 107 radiomics features extracted from each case.

| Feature type | Feature name |
| --- | --- |
| First-order statistics features (18) | original_firstorder_Energy |
|  | original_firstorder_10Percentile |
|  | original_firstorder_Variance |
|  | original_firstorder_MeanAbsoluteDeviation |
|  | original_firstorder_Median |
|  | original_firstorder_Entropy |
|  | original_firstorder_RobustMeanAbsoluteDeviation |
|  | original_firstorder_Unioriginal_formity |
|  | original_firstorder_InterquartileRange |
|  | original_firstorder_Mean |
|  | original_firstorder_TotalEnergy |
|  | original_firstorder_RootMeanSquared |
|  | original_firstorder_90Percentile |
|  | original_firstorder_Minimum |
|  | original_firstorder_Range |
|  | original_firstorder_Skewness |
|  | original_firstorder_Maximum |
|  | original_firstorder_Kurtosis |
| Shape features (14) | original_shape_MeshVolume |
|  | original_shape_VoxelVolume |
|  | original_shape_Maximum2DDiameterColumn |
|  | original_shape_MinorAxisLength |
|  | original_shape_SurfaceArea |
|  | original_shape_Maximum2DDiameterRow |
|  | original_shape_Maximum2DDiameterSlice |
|  | original_shape_SurfaceVolumeRatio |
|  | original_shape_Maximum3DDiameter |
|  | original_shape_Sphericity |
|  | original_shape_MajorAxisLength |
|  | original_shape_LeastAxisLength |
|  | original_shape_Flatness |
|  | original_shape_Elongation |
| Gray level co-occurrence matrix (GLCM) features (24) | original_glcm_ClusterProminence |
|  | original_glcm_ClusterShade |
|  | original_glcm_Id |
|  | original_glcm_Idm |
|  | original_glcm_Imc1 |
|  | original_glcm_ClusterTendency |
|  | original_glcm_DifferenceEntropy |
|  | original_glcm_DifferenceAverage |
|  | original_glcm_SumSquares |
|  | original_glcm_Contrast |
|  | original_glcm_InverseVariance |
|  | original_glcm_JointEnergy |
|  | original_glcm_DifferenceVariance |
|  | original_glcm_Imc2 |
|  | original_glcm_JointEntropy |
|  | original_glcm_SumEntropy |
|  | original_glcm_Correlation |
|  | original_glcm_MaximumProbability |
|  | original_glcm_MCC |
|  | original_glcm_Autocorrelation |
|  | original_glcm_JointAverage |
|  | original_glcm_SumAverage |
|  | original_glcm_Idmn |
|  | original_glcm_Idn |
| Gray level size zone matrix (GLSZM) features (16) | original_glszm_GrayLevelNonUniformity |
|  | original_glszm_ZoneEntropy |
|  | original_glszm_ZoneVariance |
|  | original_glszm_LargeAreaEmphasis |
|  | original_glszm_SizeZoneNonUniformity |
|  | original_glszm_ZonePercentage |
|  | original_glszm_GrayLevelNonUniformityNormalized |
|  | original_glszm_LargeAreaHighGrayLevelEmphasis |
|  | original_glszm_HighGrayLevelZoneEmphasis |
|  | original_glszm_GrayLevelVariance |
|  | original_glszm_SizeZoneNonUniformityNormalized |
|  | original_glszm_SmallAreaEmphasis |
|  | original_glszm_LargeAreaLowGrayLevelEmphasis |
|  | original_glszm_SmallAreaHighGrayLevelEmphasis |
|  | original_glszm_SmallAreaLowGrayLevelEmphasis |
|  | original_glszm_LowGrayLevelZoneEmphasis |
| Gray level run length matrix (GLRLM) features (16) | original_glrlm_RunLengthNonUniformity |
|  | original_glrlm_GrayLevelNonUniformity |
|  | original_glrlm_LongRunEmphasis |
|  | original_glrlm_RunVariance |
|  | original_glrlm_GrayLevelVariance |
|  | original_glrlm_RunEntropy |
|  | original_glrlm_ShortRunEmphasis |
|  | original_glrlm_RunLengthNonUniformityNormalized |
|  | original_glrlm_RunPercentage |
|  | original_glrlm_GrayLevelNonUniformityNormalized |
|  | original_glrlm_ShortRunHighGrayLevelEmphasis |
|  | original_glrlm_HighGrayLevelRunEmphasis |
|  | original_glrlm_LongRunHighGrayLevelEmphasis |
|  | original_glrlm_LongRunLowGrayLevelEmphasis |
|  | original_glrlm_LowGrayLevelRunEmphasis |
|  | original_glrlm_ShortRunLowGrayLevelEmphasis |
| Neighboring gray tone difference matrix (NGTDM) features (5) | original_ngtdm_Coarseness |
|  | original_ngtdm_Busyness |
|  | original_ngtdm_Complexity |
|  | original_ngtdm_Contrast |
|  | original_ngtdm_Strength |
| Gray level dependence matrix (GLDM) features (14) | original_gldm_DependenceNonUniformity |
|  | original_gldm_GrayLevelNonUniformity |
|  | original_gldm_LargeDependenceEmphasis |
|  | original_gldm_GrayLevelVariance |
|  | original_gldm_SmallDependenceEmphasis |
|  | original_gldm_DependenceEntropy |
|  | original_gldm_SmallDependenceHighGrayLevelEmphasis |
|  | original_gldm_DependenceVariance |
|  | original_gldm_DependenceNonUniformityNormalized |
|  | original_gldm_HighGrayLevelEmphasis |
|  | original_gldm_LargeDependenceHighGrayLevelEmphasis |
|  | original_gldm_LargeDependenceLowGrayLevelEmphasis |
|  | original_gldm_LowGrayLevelEmphasis |
|  | original_gldm_SmallDependenceLowGrayLevelEmphasis |

**Supplementary Data S2.**

List of the 71 radiomics features selected by the intraclass correlation coefficient screening.

| Feature type | Feature name | ICC | 95% Confidence interval |
| --- | --- | --- | --- |
| First-order | original_firstorder_Skewness^*^ | 0.998 | (0.990,0.999) |
|  | original_firstorder_Maximum^*^ | 0.984 | (0.937,0.994) |
|  | original_firstorder_Variance | 0.974 | (0.942,0.988) |
|  | original_firstorder_MeanAbsoluteDeviation | 0.970 | (0.934,0.987) |
|  | original_firstorder_Median^*^ | 0.969 | (0.906,0.988) |
|  | original_firstorder_Entropy^*^ | 0.968 | (0.929,0.986) |
|  | original_firstorder_RobustMeanAbsoluteDeviation | 0.968 | (0.929,0.986) |
|  | original_firstorder_Uniformity^*^ | 0.968 | (0.929,0.985) |
|  | original_firstorder_InterquartileRange | 0.967 | (0.928,0.985) |
|  | original_firstorder_Mean^*^ | 0.965 | (0.894,0.987) |
|  | original_firstorder_TotalEnergy^*^ | 0.961 | (0.885,0.984) |
|  | original_firstorder_RootMeanSquared^*^ | 0.956 | (0.878,0.982) |
|  | original_firstorder_90Percentile^*^ | 0.940 | (0.847,0.975) |
|  | original_firstorder_Minimum^*^ | 0.931 | (0.851,0.969) |
| Shape | original_shape_MeshVolume | 0.98 | (0.956,0.991) |
|  | original_shape_VoxelVolume | 0.979 | (0.954,0.991) |
| GLCM | original_glcm_ClusterProminence | 0.984 | (0.963,0.993) |
|  | original_glcm_ClusterShade | 0.983 | (0.961,0.992) |
|  | original_glcm_Id | 0.974 | (0.943,0.988) |
|  | original_glcm_Idmn^*^ | 0.974 | (0.942,0.988) |
|  | original_glcm_Imc1 | 0.974 | (0.943,0.989) |
|  | original_glcm_ClusterTendency | 0.972 | (0.937,0.987) |
|  | original_glcm_DifferenceEntropy | 0.972 | (0.938,0.988) |
|  | original_glcm_DifferenceAverage | 0.971 | (0.935,0.987) |
|  | original_glcm_SumSquares | 0.971 | (0.937,0.987) |
|  | original_glcm_Contrast | 0.968 | (0.929,0.986) |
|  | original_glcm_InverseVariance | 0.967 | (0.927,0.985) |
|  | original_glcm_JointEnergy | 0.966 | (0.926,0.985) |
|  | original_glcm_DifferenceVariance | 0.965 | (0.923,0.984) |
|  | original_glcm_Imc2 | 0.965 | (0.922,0.984) |
|  | original_glcm_JointEntropy^*^ | 0.962 | (0.917,0.983) |
|  | original_glcm_SumEntropy^*^ | 0.962 | (0.917,0.983) |
|  | original_glcm_Correlation | 0.958 | (0.907,0.981) |
|  | original_glcm_MaximumProbability | 0.958 | (0.906,0.981) |
|  | original_glcm_MCC | 0.938 | (0.863,0.972) |
|  | original_glcm_Autocorrelation^*^ | 0.913 | (0.805,0.961) |
| GLSZM | original_glszm_GrayLevelNonUniformity | 0.965 | (0.923,0.985) |
|  | original_glszm_ZoneEntropy | 0.957 | (0.906,0.981) |
|  | original_glszm_ZoneVariance | 0.953 | (0.897,0.979) |
|  | original_glszm_LargeAreaEmphasis | 0.952 | (0.895,0.978) |
|  | original_glszm_SizeZoneNonUniformity | 0.952 | (0.895,0.978) |
|  | original_glszm_ZonePercentage | 0.945 | (0.880,0.975) |
|  | original_glszm_GrayLevelNonUniformityNormalized | 0.941 | (0.870,0.973) |
|  | original_glszm_LargeAreaLowGrayLevelEmphasis^*^ | 0.941 | (0.865,0.974) |
|  | original_glszm_LowGrayLevelZoneEmphasis^*^ | 0.921 | (0.830,0.965) |
|  | original_SmallAreaLowGrayLevelEmphasis^*^ | 0.918 | (0.825,0.963) |
|  | original_glszm_SizeZoneNonUniformityNormalized | 0.911 | (0.810,0.960) |
| GLRLM | original_glrlm_RunLengthNonUniformity | 0.994 | (0.986,0.997) |
|  | original_glrlm_GrayLevelNonUniformity | 0.989 | (0.975,0.995) |
|  | original_glrlm_LongRunEmphasis | 0.982 | (0.961,0.992) |
|  | original_glrlm_RunVariance | 0.981 | (0.958,0.992) |
|  | original_glrlm_GrayLevelVariance | 0.979 | (0.947,0.989) |
|  | original_glrlm_RunEntropy | 0.979 | (0.953,0.991) |
|  | original_glrlm_ShortRunEmphasis | 0.978 | (0.951,0.990) |
|  | original_glrlm_RunLengthNonUniformityNormalized | 0.976 | (0.946,0.989) |
|  | original_glrlm_RunPercentage | 0.976 | (0.947,0.990) |
|  | original_glrlm_GrayLevelNonUniformityNormalized^*^ | 0.969 | (0.931,0.986) |
|  | original_glrlm_ShortRunHighGrayLevelEmphasis^*^ | 0.939 | (0.864,0.973) |
|  | original_glrlm_HighGrayLevelRunEmphasis^*^ | 0.925 | (0.834,0.966) |
| NGTDM | original_ngtdm_Coarseness | 0.994 | (0.987,0.997) |
|  | original_ngtdm_Busyness^*^ | 0.907 | (0.803,0.958) |
| GLDM | original_gldm_DependenceNonUniformity | 0.992 | (0.981,0.996) |
|  | original_gldm_GrayLevelNonUniformity | 0.979 | (0.954,0.991) |
|  | original_gldm_LargeDependenceEmphasis | 0.977 | (0.950,0.990) |
|  | original_gldm_GrayLevelVariance | 0.975 | (0.944,0.989) |
|  | original_gldm_SmallDependenceEmphasis | 0.972 | (0.937,0.987) |
|  | original_gldm_DependenceEntropy | 0.970 | (0.934,0.987) |
|  | original_gldm_SmallDependenceHighGrayLevelEmphasis | 0.963 | (0.918,0.983) |
|  | original_gldm_DependenceVariance | 0.945 | (0.880,0.975) |
|  | original_gldm_LargeDependenceHighGrayLevelEmphasis^*^ | 0.943 | (0.875,0.974) |
|  | original_gldm_SmallDependenceLowGrayLevelEmphasis^*^ | 0.919 | (0.820,0.964) |

ICC intraclass correlation coefficient, GLCM gray level co-occurrence matrix, GLSZM gray level size zone matrix, GLRLM gray level run length matrix, NGTDM neighboring gray tone difference matrix, GLDM gray level dependence matrix.

^*^the 23 radiomics features that were included in the LASSO regression analysis.
